# Volumetric Modulated Arc Therapy Planning for Craniospinal Irradiation With a New O-ring Linac

**DOI:** 10.7759/cureus.36493

**Published:** 2023-03-21

**Authors:** Kenji Matsumoto, Hajime Monzen, Kazuki Kubo, Masakazu Otsuka, Hidekazu Nambu, Yasumasa Nishimura

**Affiliations:** 1 Department of Radiology Service, Kindai University Hospital, Osakasayama, JPN; 2 Department of Medical Physics, Graduate School of Medical Sciences, Kindai University, Osaka, JPN; 3 Faculty of Medicine, Kindai University, Osakasayama, JPN

**Keywords:** auto-feathering, halcyon, rapidarc, csi, supine craniospinal irradiation

## Abstract

This study aims to determine the feasibility of using a new O-ring linear accelerator (Halcyon, Varian Medical Systems, CA, USA) to perform treatment planning using volumetric modulated arc therapy (VMAT) for craniospinal irradiation (CSI).

A 20-year-old male patient with leukemia was selected. The planning target volume (PTV) was contoured to include the entire contents of the brain and spinal canal. The PTV margin was 10 mm applied to the clinical target volume (CTV). VMAT (RapidArc, Varian Medical Systems, CA, USA) planning was performed using four isocenter with five arcs, two full rotation arcs to cover the brain and upper part of the spinal cord, and one full rotation arc for the lower part of the spinal cord. The plan was created using the auto-feathering photon optimizer calculation of the planning system. The conformity index (CI) and heterogeneity index (HI) as well as dose-volume histograms of organs at risk (OAR) were evaluated. The patient position of ±3.0 mm in the craniocaudal direction was moved in to simulate the effect of treatment inaccuracy. The total treatment time was also measured.

The CI and HI were 1.09 and 8.44, respectively. The mean dose (PTV) was 105.5%, and the mean dose (OARs) was lower than the planning dose constraints. Simulations with a patient position shift of ±3.0 mm resulted in an error of less than ±10.0% of the planned dose to the spinal cord. The total treatment time was within 15 minutes.

VMAT planning for CSI with Halcyon achieved high conformality, uniform dose distribution, low dose to the surrounding normal tissues, and reduced treatment time.

## Introduction

Craniospinal irradiation (CSI) is an important treatment method for primary tumors arising in the cranium [1]. However, CSI is difficult to engineer due to the large target volume, irregularities, and vulnerability of the spinal cord and other critical structures to radiation [2,3]. Conventional three-dimensional conformal radiation therapy (3DCRT) is still used extensively and requires matching multiple fields with different isocenters to cover the intended target volume [4]. In 3DCRT, dose inhomogeneity at the field junction is likely to occur and result in inaccuracies [3]. A common solution to minimize dose inhomogeneity is to manually move the field junctions once a week, but this method has a few drawbacks. First, because of the large dose gradient between each treatment field, even small errors in positioning can result in unintended high or low doses to the spinal cord. Second, 3DCRT CSI usually sets up the patient in the prone position to confirm the isocenter position and field junction on the skin, although the prone position is often discomforting for the patient and can cause significant patient movement at times during prolonged treatment. The prone position is restricted to the oral cavity and airway, and the position is not suitable for the immobilization of children who require paresthesia in the flexed-head position. Finally, manually shifting field junctions between fractions is complex and increases set-up errors and entire treatment time for the patient. RapidArc (RA, Varian Medical Systems, CA, USA) is a kind of volumetric modulated arc therapy (VMAT ), an intensity-modulated radiation therapy (IMRT) that modulates the multileaf collimator (MLC), dose rate, and rotational gantry speed [5,6]. For many regions and neoplasms commonly targeted by IMRT, VMAT has been compared to IMRT and has shown superiority in coverage of target, time to treatment efficiency, and preservation of organs at risk (OARs) [6-8]. Halcyon is a new linear accelerator designed to optimize RA and deliver high-quality treatment with fast image guidance. Halcyon has unique features such as a fast beam delivery with rotation, 800 MU/s dose rate, flattening filter free (FFF)-only beam, dual MLC characteristics, and daily automated image-guided radiation therapy (IGRT) workflow [9].

The purpose of this study is to establish the workflow of an RA plan for CSI cases with Halcyon to reduce treatment time, improve dose distribution of the planned target volume (PTV) and OARs, reduce potential uncertainty in beam setup, and reduce CSI sensitivity to patient motion at field junctions.

## Case presentation

**Figure 1:**
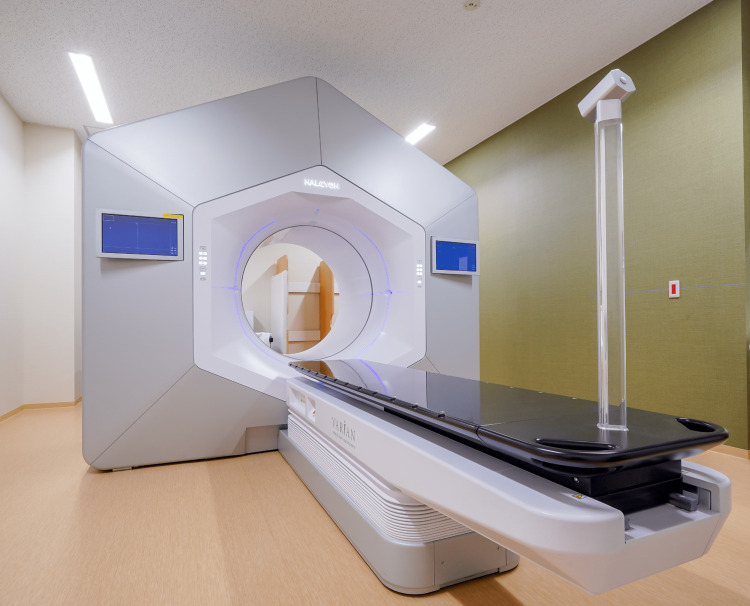
Exterior of the O-ring-type linac Halcyon.

A 20-year-old male patient (1,820 mm, 90 kg) with leukemia underwent RA CSI with Halcyon. CT datasets were acquired using a 64-slice CT scanner (GE Optima CT660; General Electric Medical Systems, Milwaukee, WI, USA), with the patient supine on a vacuum cushion (Esform; Engineering System Corporation, Nagano, Japan) in the supine position. The patient’s head and shoulders were immobilized with thermoplastic shells, and the arms were rested comfortably on the side of the body. CT images were transmitted to the Eclipse treatment planning system (version 15.6, Varian Medical Systems, Palo Alto, CA, USA) to contour the target volume and OAR for planning the subsequent treatment. Clinical target volumes (CTVs) were contoured to include the entire leptomeningeal area, cranial contents (brain and meninges), and spinal canal. A uniform 10 mm volume margin was added to the CTV to create the PTV. OAR contours were created for the lens, thyroid, esophagus, lungs, heart, liver, spleen, optic nerve, and kidneys. The prescribed dose was set at 18.0 Gy/12 fractions. The calculation dose was normalized at 95% of the PTV (D_95_). The D_max_ (maximum dose) constraint for the lens was 8.0 Gy. To account for the prescribed doses and large target volume, other doses for OARs were reduced as much as possible. Treatment was performed with 6 MV flattened filter-free photon beam irradiation from a Varian Halcyon linear accelerator (Figure 1) featuring a double-layered leaf MLC.

RA plans were created with four separate plans due to field size limitations. Patient isocenters were spaced 230 mm apart at the cerebellar, T3, T12, and L5 levels. Treatment parameters are shown in Table 1.

**Table 1:** Treatment technique parameters for craniospinal irradiation. CTV = clinical target volume; PTV = planning target volume; CCW = counterclockwise; CW = clockwise

Parameter	Patient
Age (years)/sex	20/male
Height/weight	1,820 mm/90 kg
Patient fixation	Head mask, vacuum back pillow
Length of target	838 mm
CTV to PTV margins	10 mm
Number of isocenters	4
Average distance between isocenters	230 mm
Rotation of gantry	Head: CCW and CW; Others: CCW
Angle of arc	179.0–181.0°
Angle of collimator	Head: 15.0 and 345.0°; Others: 5.0°
Average length of the overlap	55 mm
Total monitor unit	1,168.6 MU (127.4 + 132.0 + 241.8 + 327.2 + 340.2)
Prescribed dose	1.5 Gy (18 Gy/12 Fr)

**Figure 2:**
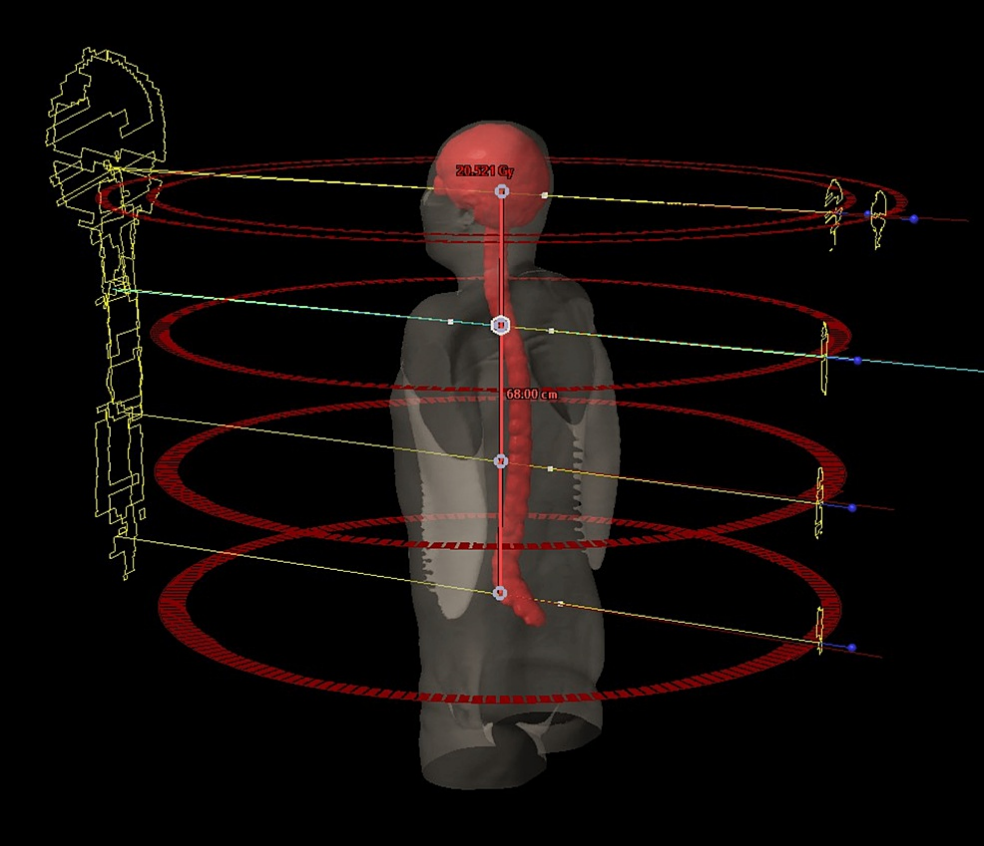
Schematic drawing of craniospinal irradiation arc delivery with four isocenters, illustrating the definitions of PTV. Arc geometry is used for the gradient optimization technique and for the overlap technique with the auto-feathering algorithm. PTV = planning target volume

To cover the upper part of the PTV (upper part of the brain and spinal cord), two coplanar arcs with opposite directions of rotation (clockwise: CW, counterclockwise: CCW) were used. A single arc was used in the other three isocenters to cover the lower part of the PTV (most of the spinal cord). In this plan, the avoidance sector in the optimization calculation was not used because it was deemed unnecessary as there were no OARs in close proximity to the PTV. Collimator angles were alternated to minimize the effect of the tongue and groove of the MLC (±15° for the head and 5° for the spinal cord). The irradiation field had an overlap of almost 60 mm. The PTV and arc placement for CSI are shown in Figure 2.

A ring-shaped control structure (inner surface 5 mm from the PTV and outer surface 15 mm) was used to enhance dose conformity and control the dose gradient outside the PTV. All arcs were optimized simultaneously, and the plan was optimized implementing dose-volume constraints on the PTV, OAR, and control structure. Further, the off-target dose was reduced using the Normal Tissue Objective (NTO) tool in Eclipse. The plan was optimized using the photon optimizer algorithm (version 15.6.03) implemented in Eclipse, and the anisotropy analysis algorithm (AAA) with a grid size of 2.5 mm was used for dose calculation (version 15.6.03).

The plans were also created using the auto-feathering calculation option of the photon optimizer [10]. This feature automatically creates a strong junction between isocenters by superimposing a linear gradient low-dose gradient between adjacent isocenter beams. Four isocenters were employed with 230 mm spacing in the craniocaudal (C-C) direction. Finally, treatment delivery using this plan required the placement of each isocenter on its plan, which required an additional CBCT imaging field. Each plan would then need to be irradiated in a separate treatment plan.

**Figure 3:**
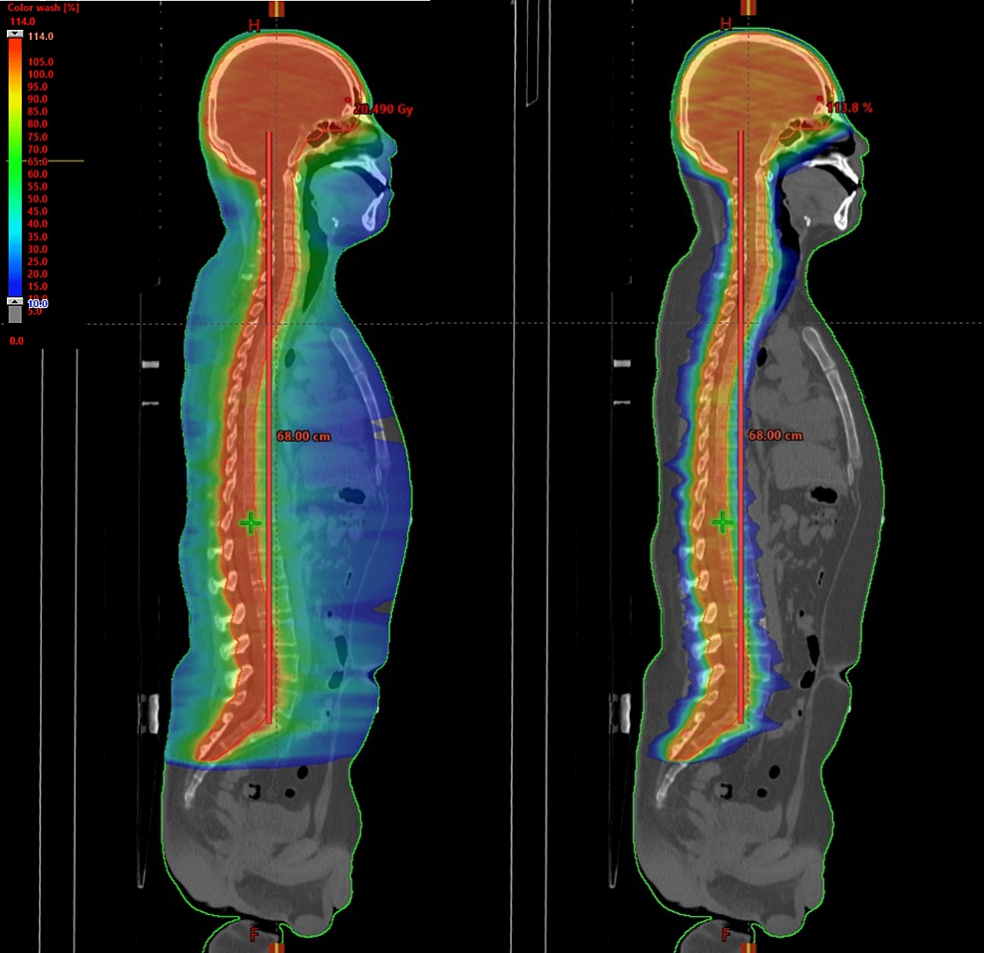
The intensity of the dose distribution in two sagittal views. Left: dose range of 10-114%. Right: dose range of 50% or more.

The composite plan was quantitatively estimated by the dose-volume histogram (DVH) analysis for the target volume and OAR. Dose parameters of average dose, D_1cc_, V_95%_, and V_110%_ for the PTV were evaluated. V_n%_ is defined as the percentage of the PTV volume that received n% of the prescribed dose. D_1cc_ is the maximum dose of 1 cm^3^ of the PTV. The conformity of the plan was measured by the conformity index (CI), defined as the ratio of the 95% isodose volume to the PTV volume of CSI (V_95%_/V_PTV_). PTV heterogeneity was measured by the homogeneity index (HI), defined as the dose received in the 5% and 95% of the PTV and the average dose received in the PTV measured as the ratio of the difference (D_5%-95%_/D_mean_). For the relevant OARs, the average dose for each organ was reported. The mean doses of the two organs were reported and compared. To further examine the quality of the RA plan, the V_95%_ of the body excluding PTV structures (i.e., normal tissue) was measured to determine if there were high-dose areas or hotspots outside of the target volume. Intentional positioning errors were also demonstrated in treatment planning to evaluate the impact of the machine imprecision and positioning errors on the interfield dose of the patient. A displacement of ±3 mm in the z-axis direction of the patient was planned. This was considered a realistic setting error, taking into account the sagging of the treatment couch, geometric calibration inaccuracies, and patient movement during delivery. Longitudinal errors were tested by moving the arc at the T3 level of the second isocenter and recalculating the dose distribution on a fixed monitor unit (MU). Treatment times were also evaluated to confirm the advantage of CSI with RA. The CSI RA plan was successfully applied to calculate dose distributions with high conformity and homogeneity. The dose distribution is shown in Figure 3.

DVH statistics for PTV and OAR are shown in Table 2.

**Table 2:** Results of calculation for 18 Gy total dose plan (1.5 Gy per fraction). PTV = planning target volume; HI = heterogeneity index; CI = conformity index; CC = craniocaudal

Traget	Parameter	Patient
PTV	D_mean_ (%)	105.5
	D_5%_ (%)	108.9
	D_95%_ (%)	100
	D_1cc_ (%)	111
	V_95%_ (%)	99.3
	V_107%_ (%)	34.8
	V_110%_ (%)	1.1
	HI	8.44
	CI	1.09
Normal tissue	V_95%_ (%)	0.5
	V_95%_ (CC)	347.4
Lens	Mean (Gy)	6.1
Thyroid	Mean (Gy)	9.3
Lung	Mean (Gy)	4.1
Liver	Mean (Gy)	3.8
Spleen	Mean (Gy)	3.3
Kidney	Mean (Gy)	4.9
Esophagus	Mean (Gy)	9.3
Left optic nerve	Mean (Gy)	19.3
Right optic nerve	Mean (Gy)	19.3

The CI and HI for this plan were 1.09 and 8.44, respectively. The average PTV dose was 105.5%, the V_110%_ was less than 2%, and the V_107%_ was 34.8%. The CSI RA method also achieved tolerable OAR doses. The mean doses to the lens, lung, heart, spleen, kidney, optic nerves, and normal tissue are shown in Table 2.

A simulated longitudinal position error of ±3.0 mm in the plan resulted in a maximum dose error of 114.11% and a minimum dose error of 98.26% for the PTV. The corresponding arc junction dose error was within ±10%. The total treatment time for CSI RA was less than 15 minutes, allowing the irradiation to be performed rapidly.

## Discussion

In this study, CSI was performed in the supine position using Halcyon RA. The results show that the RA multi-isocenter method can achieve a uniform and conformal dose to the target while simultaneously constraining the dosage to the associated OARs. The Halcyon system, by using overlapping arcs and 3D image guidance with CBCT, can be used to reduce difficulty in matching field junctions. Lee et al. developed a VMAT technique using smart arcs for CSI (23.4 Gy/13 fractions) and compared it to conventional radiation [11]. They reported significantly lower mean and maximum doses to the heart, thyroid, esophagus, optic nerves, and eye with median values of 1.22 Gy (1.09-1.45) and 1.04 Gy (1.03-1.07), respectively. In general, VMAT produces a high uniform dose distribution due to the high angular sampling rate of the radiation beam. Parameter variability depends on the weighting and prioritization used during planning. Conventional IMRT and tomotherapy have also been studied in CSI [3,12-14]. Seppälä et al. used a single plan with dynamic split-field IMRT to improve the target dose uniformity in CSI [3]. Results showed that split-field IMRT improved target volume dose uniformity compared to conventional 3DCRT. Sharma et al. compared 3DCRT, IMRT, and tomotherapy in the treatment of CSI in children and adolescents [12]. These techniques were compared dosimetrically. The study concluded that tomotherapy is technically easier and would have a dosimetric advantage over alternative methods. Major problems with tomotherapy and IMRT have been the increase in total cumulative MU and prolonged treatment time. These two issues further led to concerns about intrafraction setup errors. In this study, we found that Halcyon RA was able to deliver a high conformal dose distribution equivalent to the helical tomotherapy method with fewer MUs, as shown in Table 1.

In CSI therapy, mechanical precision is essential, especially when a single plan delivers the entire dose at a high gradient. Typical mechanical tolerances are about ±1 mm for the MLC position, couch, and isocenter movement during gantry rotation. When considering technical uncertainties, the patient’s motion must also be taken into account. With Halcyon, position verification and correction via IGRT (CBCT imaging) in each plan made accurate beam delivery possible, and the total treatment time with IGRT was less than 15 minutes.

**Figure 4:**
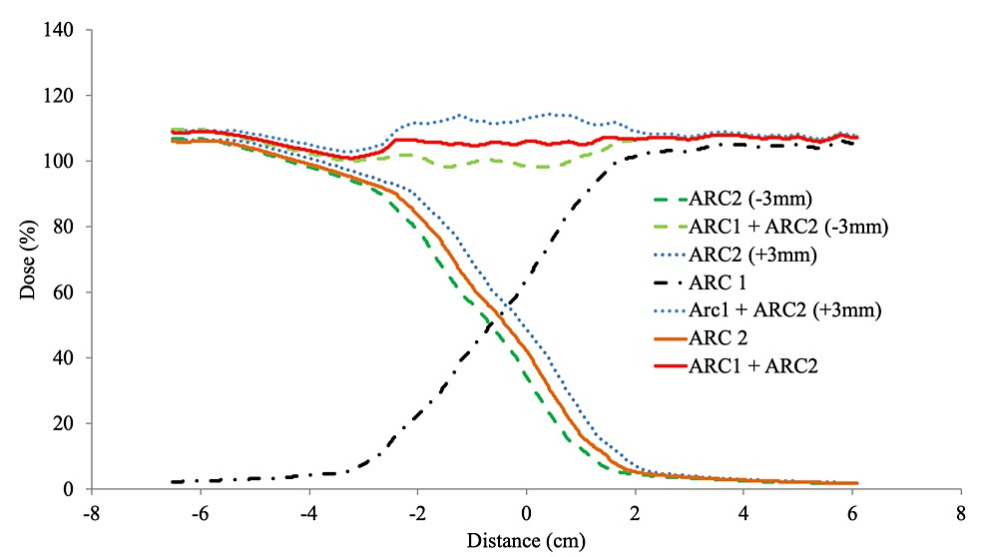
The results of the dose-accumulation simulation with longitudinal positional error. Arc2 in the cervical region was moved with a position error of ±3 mm in the superior-inferior direction.

In this study, the dose error due to displacement of ±3 mm in the C-C direction was verified. It was shown that auto-feathering generated a gradual dose gradient at the overlapping area of each arc (Figure 4).

The positional error of 3 mm in the longitudinal direction, which was a concern in the conventional irradiation method, had little effect on the dose distribution, resulting in a dose difference of less than ±10% of the spinal cord dose calculation value. Because the field-matching error was random and IGRT with CBCT was performed at each isocenter, it is safe to assume that the field-matching error was less than 3 mm. Thus, the overall dose uncertainty in CSI treatment of RA is expected to be much lower than the reported 10%. This result was obtained by using functional auto-feathering in RA optimization. CSI is concerned with secondary malignancies, especially in pediatric patients. RA can reduce doses to the thyroid, larynx, heart, and esophagus compared to conventional 3DCRT techniques. On the other hand, an expansion of the low-dose area occurred in the RA treatment plan. It is not yet clear the biological effects that the increased volume at low doses can have. Given the risk of secondary malignancies, it is currently unclear whether intermediate doses are safe for multiple organs. The dose to normal organs surrounding the PTV occurs primarily through transmission from the linear accelerator head, and this dose is proportional to the number of MUs. In general, RA methods are designed to use MUs more efficiently than conventional IMRT. MU of the CSI RA plan in this study was as low as that of 3DCRT and much less than that of conventional IMRT or helical tomotherapy [7]. Thus, RA may play a role in reducing the potential for the development of secondary cancers in CSI and in other large treatment sites [15]. A further major advantage of Halcyon RA compared to more labor-intensive treatments such as conventional IMRT is the ease with which the treatment can be performed. The time required to perform the treatment is reduced by 10-20 minutes compared to IMRT or tomotherapy [12].

## Conclusions

This study reports a practical and effective treatment plan for CSI RA with Halcyon. Compared to conventional 3DCRT, Halcyon RA achieved high conformality and uniform dose distribution, as well as low dose to the surrounding normal tissue, making it safer for the patient and reducing treatment time. In addition, optimization with auto-feathering was effective in reducing uncertainty due to mechanical inaccuracies and patient setup errors, making patient treatment safer. Future CSI treatments with Halcyon RA will benefit patients.
